# Supplementary material for: Obligate mutualistic cooperation limits evolvability
Source: Nat Commun. 2022 Jan 17;13:337. doi: 10.1038/s41467-021-27630-9 (PMC8764027; doi:10.1038/s41467-021-27630-9)
Supplement: Supplementary file 1 — Supplementary information [file 41467_2021_27630_MOESM1_ESM.pdf]

Supplementary information

Obligate mutualistic cooperation limits evolvability

Benedikt Pauli[1], Leonardo Oña[1], Marita Herman[1,2], Christian Kost[1]*

[1] *Department of Ecology, Osnabrück University, Barbarastraße 13, 49076 Osnabrück, Germany*

[2] *Present address: Department of Plant Physiology, Osnabrück University, Barbarastr. 11, 49076 Osnabrück, Germany*

* Email: christiankost@gmail.com

Table of contents **Page**

Supplementary Figures 2

Supplementary Tables 8

Supplementary Note 15

Supplementary References 16

Supplementary Figures

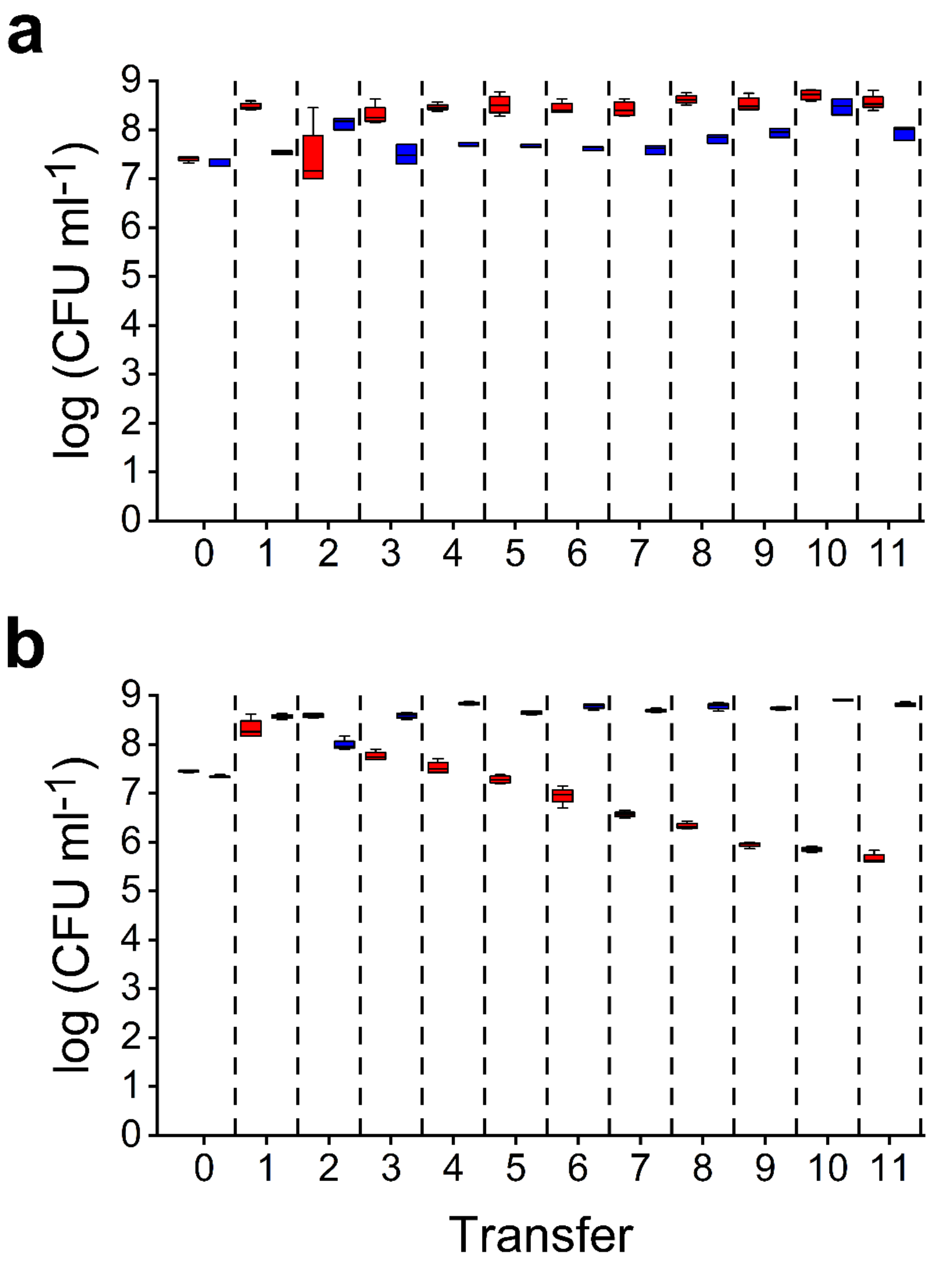

Supplementary Figure 1 | Cocultures of auxotrophs reach a stable equilibrium in the absence, but not the presence of externally provided amino acids. Cocultures of *E. coli* BW25113 *ΔtrpB ara*$^-$ Δ*lacZ* (TRP, red boxes) and *E. coli* BW25113 *ΔtyrA ara*$^+$ *lacZ*$^+$ (TYR, blue boxes) were serially propagated every 72 h **(a)** without and **(b)** with amino acid supplementation (100 µM each). Shown are the numbers of colony-forming units (CFU) of each strain per millilitre of culture. Boxes show median values (horizontal line in boxes) and the upper and lower quartiles (i.e. 25-75% of data, boxes). Whiskers indicate the 1.5x interquartile range (n = 4). Source data are provided as a Source Data file.

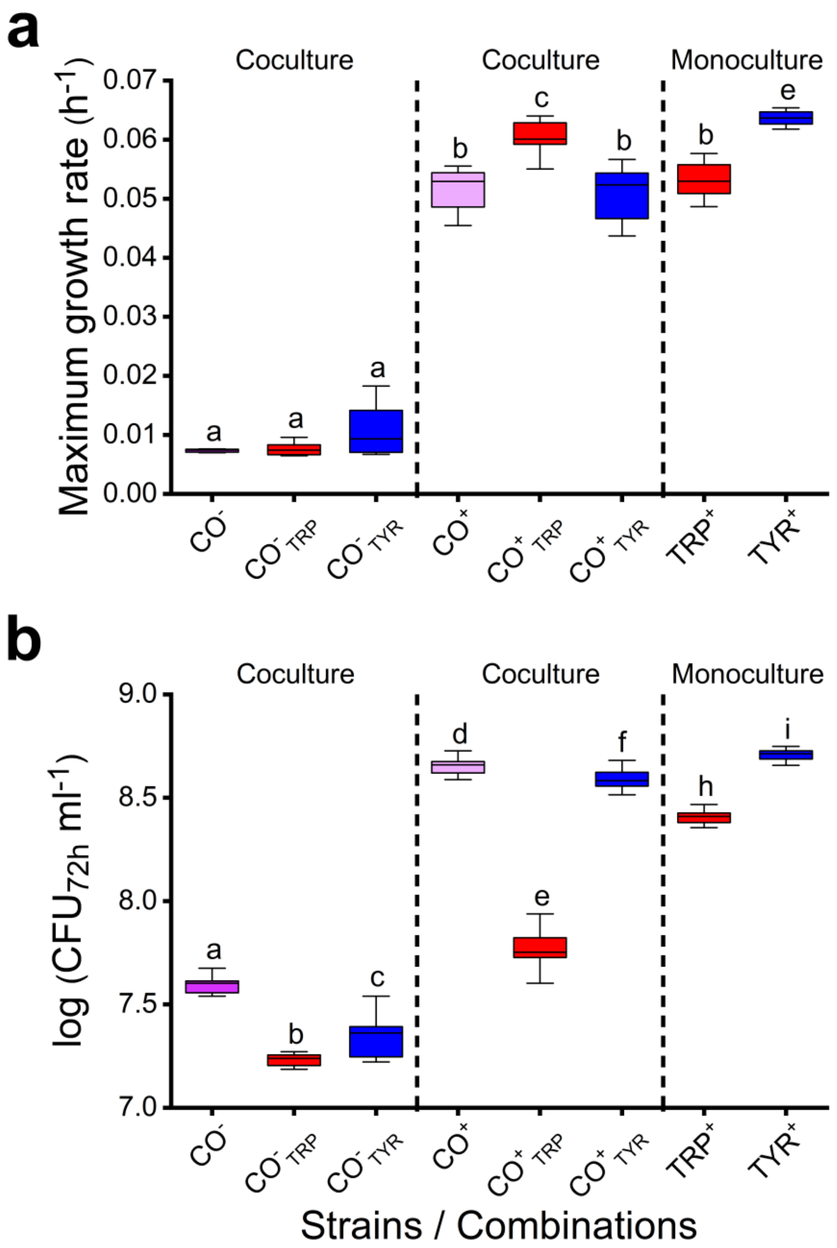

Supplementary Figure 2 | Growth of ancestral populations depends on amino acid supplementation. (a) Maximum growth rate and **(b)** final yield after 72 h of growth of cocultures (CO, purple boxes), individual genotypes within cocultures (CO_{TRP} and CO_{TYR}) or monocultures of *E. coli* BW25113 *ΔtrpB ara*$^{-}$ Δ*lacZ* (TRP, red boxes) and *E. coli* BW25113 *ΔtyrA ara*$^{+}$ *lacZ*$^{+}$ (TYR, blue boxes). Populations were grown in minimal medium with ($^{+}$) or without ($^{-}$) amino acid supplementation and growth parameters were determined by quantifying the number of colony-forming units (CFUs). Box plots display median values (horizontal line in boxes) and the upper and lower quartiles (i.e. 25-75% of data, boxes). Whiskers show the 1.5x interquartile range. Different letters above boxes indicate significant differences between strains (two-sided Mann-Whitney-U-Test followed by Benjamini-Hochberg correction: $P < 0.05$, $n = 8$). For exact P-values, see Supplementary Table 1. Source data are provided as a Source Data file.

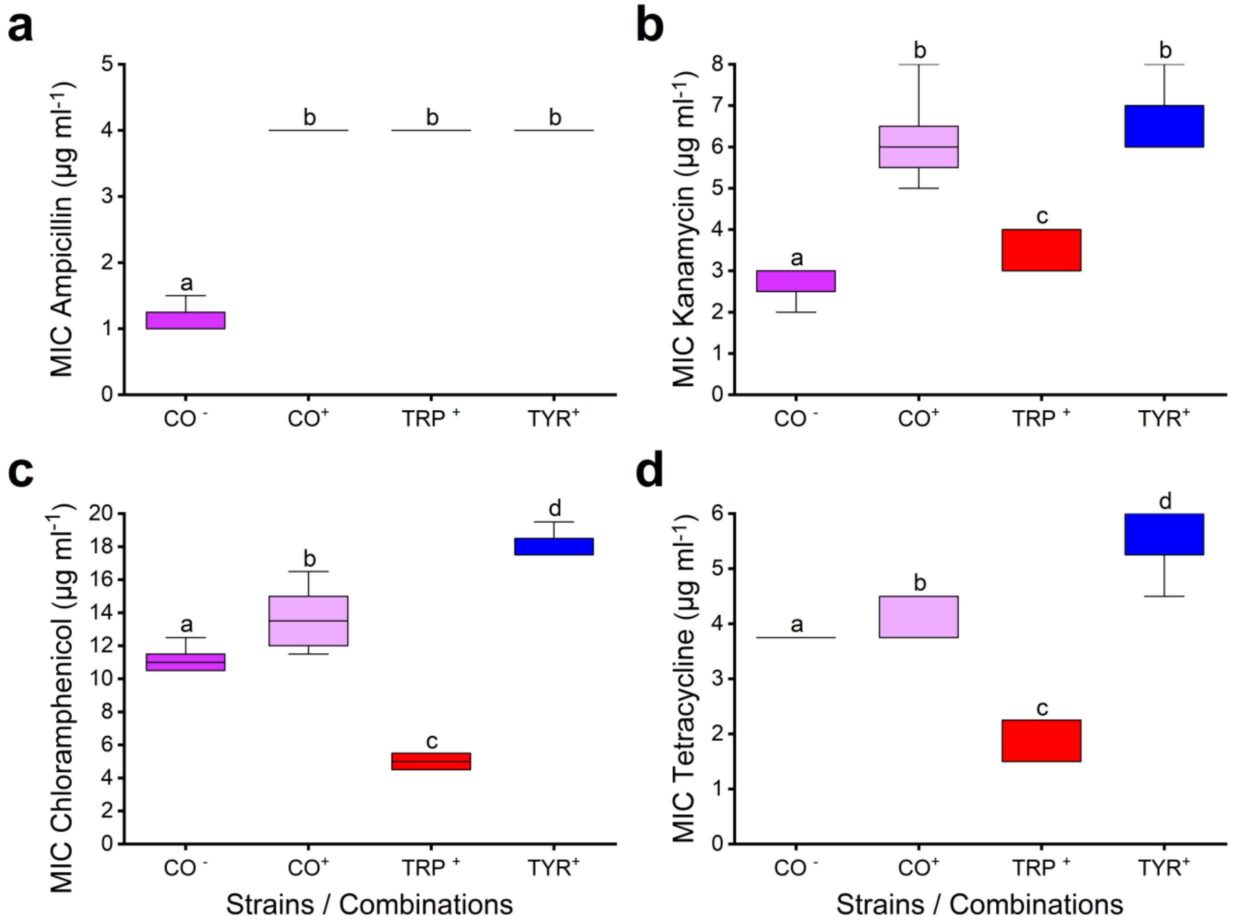

Supplementary Figure 3 | Ancestral populations differ in their minimal inhibitory concentrations (MICs). Cocultures (CO, purple boxes) and monocultures of *E. coli* BW25113 *ΔtrpB ara*$^{-}$ Δ*lacZ* (TRP, red boxes) and *E. coli* BW25113 *ΔtyrA ara*$^{+}$ *lacZ*$^{+}$ (TYR, blue boxes) were grown in minimal medium supplemented with increasing concentrations of the antibiotic **(a)** ampicillin, **(b)** kanamycin, **(c)** chloramphenicol, and **(d)** tetracycline. Cultures were grown with ($^{+}$) or without ($^{-}$) amino acid supplementation and growth was evaluated by measuring the optical density (OD_{600nm}). Cultures' MIC was defined as the first concentration were the OD_{600nm} value did not exceed a threshold of 0.01. Box plots show median values (horizontal line in boxes) and the upper and lower quartiles (i.e. 25-75% of data, boxes). Whiskers represent the 1.5x interquartile range. Different letters above box plots indicate significant differences between groups (two-sided Mann-Whitney-U-Test followed by Benjamini-Hochberg correction: $P < 0.05$, $n = 8$). For exact P-values, see Supplementary Table 2. Source data are provided as a Source Data file.

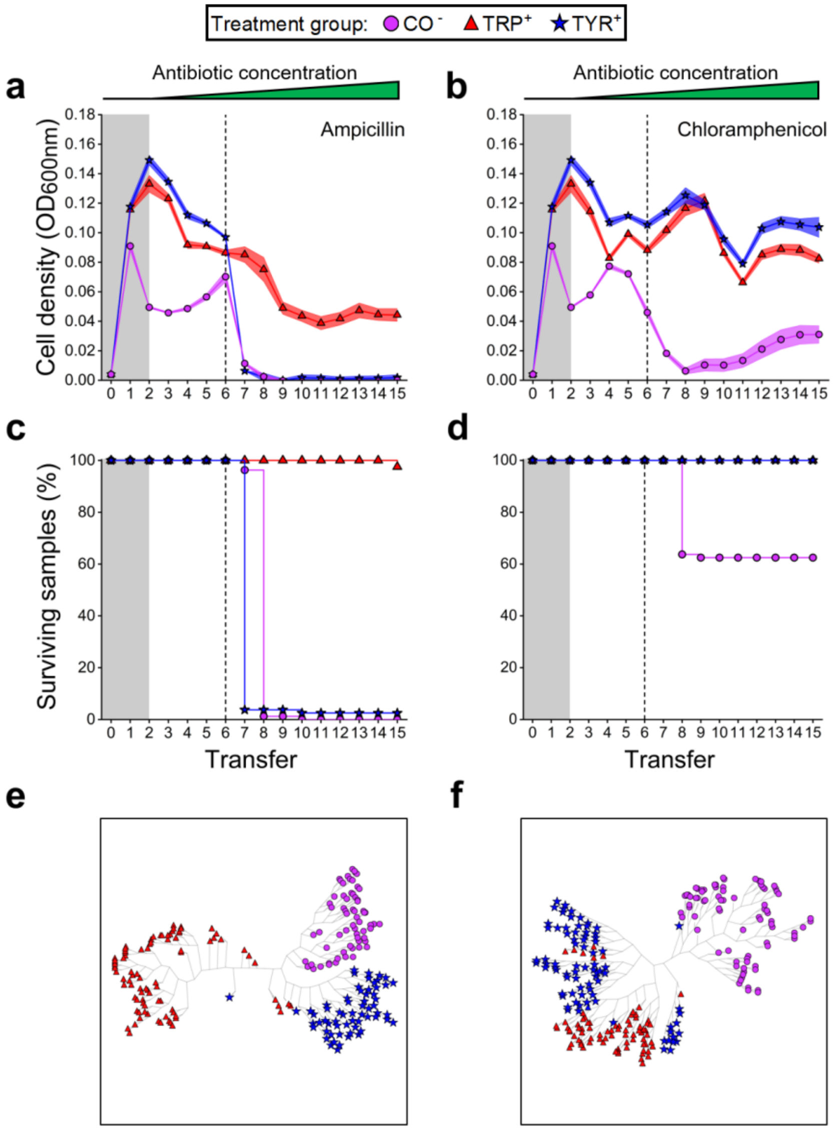

Supplementary Figure 4 | Effect of the ampicillin and chloramphenicol treatment on the growth, survival, and evolutionary trajectory of experimental populations. (a, b) Mean growth (±95% confidence interval, n = 80 per point) quantified as OD_{600nm} and **(c, d)** proportion of surviving replicates in percent (n = 80 per strain) of auxotrophic monocultures (TRP, TYR) and mutualistic cocultures (CO, purple circles) of the tryptophan (TRP, red triangles) and tyrosine (TYR, blue star) auxotrophic strains throughout the evolution experiment. Antibiotic concentrations were increased in a stepwise manner after each transfer (i.e. every 72 h) (Supplementary Fig. 5). Grey-shaded areas indicate periods without antibiotic treatment. The green triangles above represent the increasing antibiotic concentrations in the evolution experiment (a-d). **(a, c, e)** ampicillin treatment **(b, d, f)** chloramphenicol treatment. Dashed lines mark the point, at which antibiotic concentrations exceeded sub-MIC values. Monocultures were supplemented ($^{+}$) with both amino acids (100 µM each), while cocultured bacteria depended on the amino acids provided by their respective cross-feeding partner ($^{-}$). **(e, f)** Clustering trees of cell density profiles of experimental cultures across transfers indicate differences in the evolutionary trajectories taken by the different populations. Each leaf within a given tree represents a replicate (n = 80 per strain). A radial embedding layout was used to display trees. Source data are provided as a Source Data file.

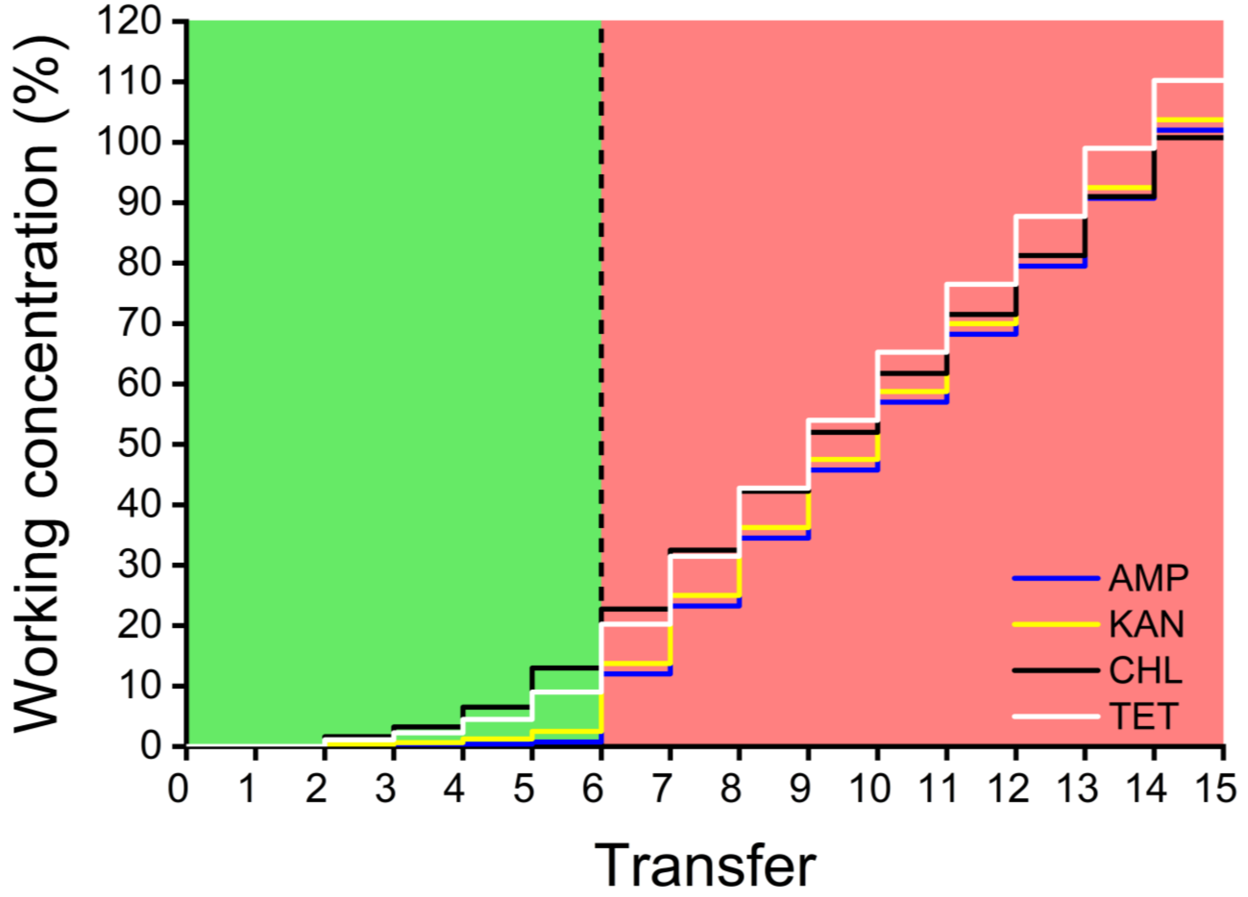

Supplementary Figure 5 | Ramping of antibiotic concentrations during the evolution experiment. Concentrations of antibiotics displayed in percent of the respective working concentration (i.e. ampicillin: 100 µg ml^{-1} (AMP), kanamycin: 50 µg ml^{-1} (KAN), chloramphenicol: 25 µg ml^{-1} (CHL), and tetracycline: 15 µg ml^{-1} (TET)). The green area represents antibiotic concentrations below and the red area above the determined sub-minimal inhibitory concentrations (sub-MIC). Antibiotic concentrations doubled at each transfer, starting from the second transfer. Cultures were transferred every 72 h to fresh medium. Source data are provided as a Source Data file.

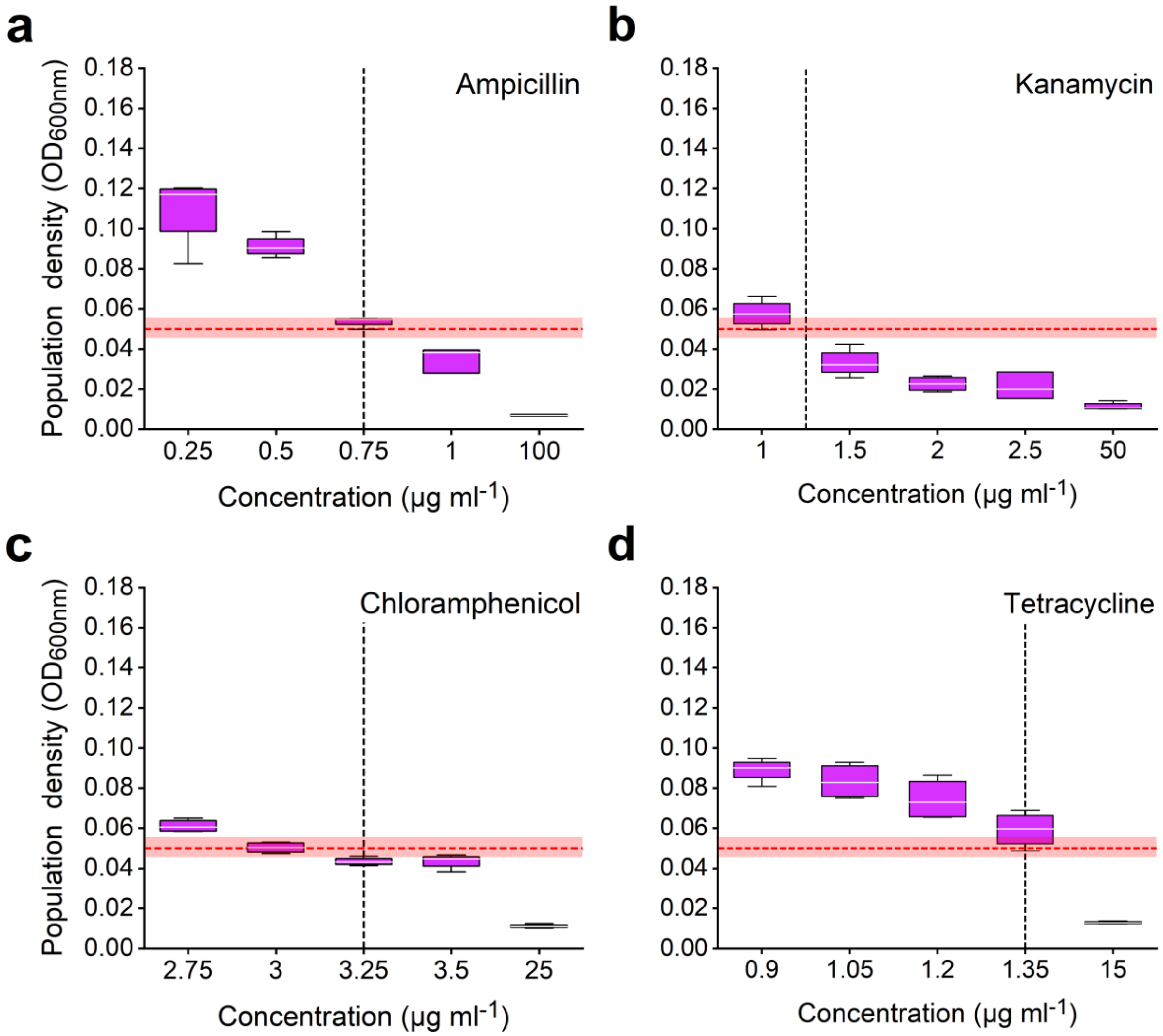

Supplementary Figure 6 | Determination of sub-minimal inhibitory concentrations (sub-MIC) for populations of cocultured auxotrophs. Cocultures of *E. coli* BW25113 *ΔtrpB ara*$^-$ Δ*lacZ* and *E. coli* BW 25113 *ΔtyrA ara*$^+$ *lacZ*$^+$ (purple boxes) were grown in minimal medium supplemented with increasing concentrations of the antibiotic **(a)** ampicillin, **(b)** kanamycin, **(c)** chloramphenicol, and **(d)** tetracycline. Growth was evaluated by measuring the optical density (OD_{600nm}) of cultures. The red dashed line and the red-shaded area represent the threshold zone (0.05 ± 0.005 OD_{600nm}). The black dashed line indicates the sub-MIC. Boxes show median values (horizontal line in boxes) and the upper and lower quartiles (i.e. 25-75% of data, boxes). Whiskers indicate the 1.5x interquartile range (n = 4). Source data are provided as a Source Data file.

Supplementary Tables

Supplementary Table 1 | Statistical results of comparisons shown in Supplementary Figure 2. Experimental strains that were part of a coculture are indicated as CO. Subscripted auxotrophies (i.e. TRP or TYR) represent individual constituent genotypes and superscript signs (i.e. $^{+}$ or $^{-}$) indicate the absence and presence of amino acid supplementation. P-values were determined by two-sided Mann-Whitney-U-Test and have been corrected using the Benjamini-Hochberg procedure (P_{adj}). See Supplementary Table 5 for abbreviations of experimental groups.

Figure	Group 1	Group 2	P_{adj}	W	n
Sup. 2 a	CO^{-}	CO^{-}_{TRP}	0.878	30	8
Sup. 2 a	CO^{-}	CO^{-}_{TYR}	0.274	20	8
Sup. 2 a	CO^{-}	CO^{+}	< 0.001	0	8
Sup. 2 a	CO^{-}	CO^{+}_{TRP}	< 0.001	0	8
Sup. 2 a	CO^{-}	CO^{+}_{TYR}	< 0.001	0	8
Sup. 2 a	CO^{-}	TRP^{+}	< 0.001	0	8
Sup. 2 a	CO^{-}	TYR^{+}	< 0.001	0	8
Sup. 2 a	CO^{-}_{TRP}	CO^{-}_{TYR}	0.195	18	8
Sup. 2 a	CO^{-}_{TRP}	CO^{+}	< 0.001	0	8
Sup. 2 a	CO^{-}_{TRP}	CO^{+}_{TRP}	< 0.001	0	8
Sup. 2 a	CO^{-}_{TRP}	CO^{+}_{TYR}	< 0.001	0	8
Sup. 2 a	CO^{-}_{TRP}	TRP^{+}	< 0.001	0	8
Sup. 2 a	CO^{-}_{TRP}	TYR^{+}	< 0.001	0	8
Sup. 2 a	CO^{-}_{TYR}	CO^{+}	< 0.001	0	8
Sup. 2 a	CO^{-}_{TYR}	CO^{+}_{TRP}	< 0.001	0	8
Sup. 2 a	CO^{-}_{TYR}	CO^{+}_{TYR}	< 0.001	0	8
Sup. 2 a	CO^{-}_{TYR}	TRP^{+}	< 0.001	0	8
Sup. 2 a	CO^{-}_{TYR}	TYR^{+}	< 0.001	0	8
Sup. 2 a	CO^{+}	CO^{+}_{TRP}	< 0.001	1	8
Sup. 2 a	CO^{+}	CO^{+}_{TYR}	0.878	34	8
Sup. 2 a	CO^{+}	TRP^{+}	0.618	26	8
Sup. 2 a	CO^{+}	TYR^{+}	< 0.001	0	8
Sup. 2 a	CO^{+}_{TRP}	CO^{+}_{TYR}	< 0.001	63	8
Sup. 2 a	CO^{+}_{TRP}	TRP^{+}	< 0.001	62	8
Sup. 2 a	CO^{+}_{TRP}	TYR^{+}	0.026	10	8
Sup. 2 a	CO^{+}_{TYR}	TRP^{+}	0.566	25	8
Sup. 2 a	CO^{+}_{TYR}	TYR^{+}	< 0.001	0	8
Sup. 2 a	TRP^{+}	TYR^{+}	< 0.001	0	8
Sup. 2 b	CO^{-}	CO^{-}_{TRP}	0.001	64	8
Sup. 2 b	CO^{-}	CO^{-}_{TYR}	0.001	63.5	8

Sup. 2 b	CO^-	CO^+	0.001	0	8
Sup. 2 b	CO^-	CO^+_{TRP}	0.004	4	8
Sup. 2 b	CO^-	CO^+_{TYR}	0.001	0	8
Sup. 2 b	CO^-	TRP^+	0.001	0	8
Sup. 2 b	CO^-	TYR^+	0.001	0	8
Sup. 2 b	CO^-_{TRP}	CO^-_{TYR}	0.036	11.5	8
Sup. 2 b	CO^-_{TRP}	CO^+	0.001	0	8
Sup. 2 b	CO^-_{TRP}	CO^+_{TRP}	0.001	0	8
Sup. 2 b	CO^-_{TRP}	CO^+_{TYR}	0.001	0	8
Sup. 2 b	CO^-_{TRP}	TRP^+	0.001	0	8
Sup. 2 b	CO^-_{TRP}	TYR^+	0.001	0	8
Sup. 2 b	CO^-_{TYR}	CO^+	0.001	0	8
Sup. 2 b	CO^-_{TYR}	CO^+_{TRP}	0.001	0	8
Sup. 2 b	CO^-_{TYR}	CO^+_{TYR}	0.001	0	8
Sup. 2 b	CO^-_{TYR}	TRP^+	0.001	0	8
Sup. 2 b	CO^-_{TYR}	TYR^+	0.001	0	8
Sup. 2 b	CO^+	CO^+_{TRP}	0.001	64	8
Sup. 2 b	CO^+	CO^+_{TYR}	0.040	52	8
Sup. 2 b	CO^+	TRP^+	0.001	64	8
Sup. 2 b	CO^+	TYR^+	0.019	9	8
Sup. 2 b	CO^+_{TRP}	CO^+_{TYR}	0.001	0	8
Sup. 2 b	CO^+_{TRP}	TRP^+	0.001	0	8
Sup. 2 b	CO^+_{TRP}	TYR^+	0.001	0	8
Sup. 2 b	CO^+_{TYR}	TRP^+	0.001	64	8
Sup. 2 b	CO^+_{TYR}	TYR^+	0.002	1	8
Sup. 2 b	TRP^+	TYR^+	0.001	0	8

Supplementary Table 2 | Statistical results of comparisons shown in Supplementary Figure 3. The superscript signs (i.e. $^{+}$ or $^{-}$) indicate the absence and presence of amino acid supplementation. Dashes indicate comparisons with no measurable difference, because all replicates within both groups reached identical values. P-values were determined by two-sided Mann-Whitney-U-Test and have been corrected using the Benjamini-Hochberg procedure (P_{adj}). See Supplementary Table 5 for abbreviations of experimental groups.

Figure	Group 1	Group 2	P_{adj}	W	n
Sup. 3 a	CO^{-}	CO^{+}	< 0.001	0	8
Sup. 3 a	CO^{-}	TRP^{+}	< 0.001	0	8
Sup. 3 a	CO^{-}	TYR^{+}	< 0.001	0	8
Sup. 3 a	CO^{+}	TRP^{+}	-	-	8
Sup. 3 a	CO^{+}	TYR^{+}	-	-	8
Sup. 3 a	TRP^{+}	TYR^{+}	-	-	8
Sup. 3 b	CO^{-}	CO^{+}	0.001	0	8
Sup. 3 b	CO^{-}	TRP^{+}	0.041	15	8
Sup. 3 b	CO^{-}	TYR^{+}	0.001	0	8
Sup. 3 b	CO^{+}	TRP^{+}	0.001	64	8
Sup. 3 b	CO^{+}	TYR^{+}	0.352	23.5	8
Sup. 3 b	TRP^{+}	TYR^{+}	0.001	0	8
Sup. 3 c	CO^{-}	CO^{+}	< 0.001	10.5	8
Sup. 3 c	CO^{-}	TRP^{+}	0.001	64	8
Sup. 3 c	CO^{-}	TYR^{+}	0.001	0	8
Sup. 3 c	CO^{+}	TRP^{+}	0.001	64	8
Sup. 3 c	CO^{+}	TYR^{+}	0.003	2.5	8
Sup. 3 c	TRP^{+}	TYR^{+}	0.002	0	8
Sup. 3 d	CO^{-}	CO^{+}	0.005	5.5	8
Sup. 3 d	CO^{-}	TRP^{+}	< 0.001	64	8
Sup. 3 d	CO^{-}	TYR^{+}	< 0.001	0	8
Sup. 3 d	CO^{+}	TRP^{+}	< 0.001	64	8
Sup. 3 d	CO^{+}	TYR^{+}	< 0.001	0	8
Sup. 3 d	TRP^{+}	TYR^{+}	< 0.001	0	8

Supplementary Table 3 | Log-rank test results of the survival analysis. Dashes indicate comparisons with no measurable difference, because all replicates within both groups survived until the end of the evolution experiment. See Supplementary Table 5 for abbreviations of experimental groups.

Figure	Treatment	Group 1	Group 2	P	χ^2	n
2 c	Kanamycin	CO	TRP	$1.3 * 10^{-33}$	145.95	80
2 c	Kanamycin	CO	TYR	$2.1 * 10^{-27}$	117.6	80
2 c	Kanamycin	TRP	TYR	0.16	1.97	80
2 d	Tetracycline	CO	TRP	$1.9 * 10^{-16}$	67.71	80
2 d	Tetracycline	CO	TYR	$1.9 * 10^{-16}$	67.71	80
2 d	Tetracycline	TRP	TYR	-	-	80
Sup. 4 c	Ampicillin	CO	TRP	$1.1 * 10^{-36}$	160.02	80
Sup. 4 c	Ampicillin	CO	TYR	$2.3 * 10^{-25}$	108.34	80
Sup. 4 c	Ampicillin	TRP	TYR	$3.2 * 10^{-35}$	153.35	80
Sup. 4 d	Chloramphenicol	CO	TRP	$1.3 * 10^{-9}$	36.76	80
Sup. 4 d	Chloramphenicol	CO	TYR	$1.3 * 10^{-9}$	36.76	80
Sup. 4 d	Chloramphenicol	TRP	TYR	-	-	80

Supplementary Table 4 | Statistical results of comparisons shown in Figure 3. Cocultures are indicated as CO. Monocultured auxotrophs (i.e. TRP or TYR) had two distinct evolutionary backgrounds specified as coevolved ($_{CO}$) and monoevolved ($_{MO}$). Superscript signs (i.e. $^{+}$ or $^{-}$) indicate the absence and presence of amino acid supplementation. P-values were determined by applying a two-sided Mann-Whitney-U-Test and have been corrected using the Benjamini-Hochberg procedure (P_{adj}). See Supplementary Table 5 for abbreviations of experimental groups.

Figure	Group 1	Group 2	P_{adj}	W	n
3 a	CO^-	CO^+	0.088	49	8
3 a	CO^-	TRP^+_{CO}	0.003	62	8
3 a	CO^-	TYR^+_{CO}	0.003	64	8
3 a	CO^-	TRP^+_{MO}	0.004	3	8
3 a	CO^-	TYR^+_{MO}	0.003	0	8
3 a	CO^+	TRP^+_{CO}	0.062	51	8
3 a	CO^+	TYR^+_{CO}	0.003	62	8
3 a	CO^+	TRP^+_{MO}	0.007	5	8
3 a	CO^+	TYR^+_{MO}	0.008	4	8
3 a	TRP^+_{CO}	TYR^+_{CO}	0.233	44	8
3 a	TRP^+_{CO}	TRP^+_{MO}	0.003	0	8
3 a	TRP^+_{CO}	TYR^+_{MO}	0.003	0	8
3 a	TYR^+_{CO}	TRP^+_{MO}	0.003	0	8
3 a	TYR^+_{CO}	TYR^+_{MO}	0.003	0	8
3 a	TRP^+_{MO}	TYR^+_{MO}	0.684	24	8
3 b	CO^-	CO^+	0.004	59	8
3 b	CO^-	TRP^+_{CO}	< 0.001	0	8
3 b	CO^-	TYR^+_{CO}	< 0.001	256	8 and 32
3 b	CO^-	TRP^+_{MO}	0.001	0	8
3 b	CO^-	TYR^+_{MO}	0.004	59	8
3 b	CO^+	TRP^+_{CO}	< 0.001	0	8
3 b	CO^+	TYR^+_{CO}	< 0.001	256	8 and 32
3 b	CO^+	TRP^+_{MO}	< 0.001	0	8
3 b	CO^+	TYR^+_{MO}	0.005	57	8
3 b	TRP^+_{CO}	TYR^+_{CO}	< 0.001	256	8 and 32
3 b	TRP^+_{CO}	TRP^+_{MO}	0.006	7	8
3 b	TRP^+_{CO}	TYR^+_{MO}	< 0.001	64	8
3 b	TYR^+_{CO}	TRP^+_{MO}	< 0.001	0	8 and 32
3 b	TYR^+_{CO}	TYR^+_{MO}	0.004	52.5	8
3 b	TRP^+_{MO}	TYR^+_{MO}	< 0.001	64	8

Supplementary Table 5 | Strains and abbreviations.

Abbreviation	Strain
TRP	*E. coli* BW25113 Δ*trpB ara*$^-$ Δ*lacZ*
TYR	*E. coli* BW 25113 Δ*tyrA ara*$^+$ *lacZ*$^+$
CO	*E. coli* BW25113 Δ*trpB ara*$^-$ Δ*lacZ* cocultured with *E. coli* BW 25113 Δ*tyrA ara*$^+$ *lacZ*$^+$
CO_{TRP}	Separated data from *E. coli* BW25113 Δ*trpB ara*$^-$ Δ*lacZ* in coculture
CO_{TYR}	Separated data from *E. coli* BW 25113 Δ*tyrA ara*$^+$ *lacZ*$^+$ in coculture
CO_{EVO}	Coevolved *E. coli* BW25113 Δ*trpB ara*$^-$ Δ*lacZ* and coevolved *E. coli* BW 25113 Δ*tyrA ara*$^+$ *lacZ*$^+$
CO_{AUX}	Monoevolved *E. coli* BW25113 Δ*trpB ara*$^-$ Δ*lacZ* and monoevolved *E. coli* BW 25113 Δ*tyrA ara*$^+$ *lacZ*$^+$ in coculture

Supplementary Table 6 | Statistical results of comparisons shown in Figure 4. Cocultures of previously coevolved auxotrophs (CO_{EVO}) and of previously monoevolved auxotrophs (CO_{AUX}) are compared. Superscript signs (i.e. $^{+}$ or $^{-}$) indicate the absence and presence of amino acid supplementation. Comparisons were performed using a linear mixed model (Fig. 4a,b,d,e) and applying a two-sided Mann-Whitney-U-Test followed by the Benjamini-Hochberg correction (Fig. 4c,f). Data corresponding to the Mann-Whitney-U-Tests are marked with a star (★). See Supplementary Table 5 for abbreviations of experimental groups and the material and methods part for a more details description.

Figure	Treatment	Strain 1	Strain 2	P (P_{adj}*)	t ratio (W*)	n
4 a	Chloramphenicol	CO_{EVO}^{-}	CO_{AUX}^{-}	< 0.001	- 5.71	8
4 b	Chloramphenicol	CO_{EVO}^{+}	CO_{AUX}^{+}	< 0.001	- 7.42	8
4 a,b	Chloramphenicol	CO_{EVO}^{-}	CO_{EVO}^{+}	0.0675	2.637	8
4 a,b	Chloramphenicol	CO_{AUX}^{-}	CO_{AUX}^{+}	< 0.001	- 15.93	8
4 c	Chloramphenicol	CO_{EVO}^{-}	CO_{AUX}^{-}	0.2388★	96★	16★
4 c	Chloramphenicol	CO_{EVO}^{-}	CO_{EVO}^{+}	< 0.001★	15★	16★
4 c	Chloramphenicol	CO_{EVO}^{-}	CO_{AUX}^{+}	< 0.001★	7★	16★
4 c	Chloramphenicol	CO_{AUX}^{-}	CO_{EVO}^{+}	< 0.001★	20★	16★
4 c	Chloramphenicol	CO_{AUX}^{-}	CO_{AUX}^{+}	< 0.001★	3★	16★
4 c	Chloramphenicol	CO_{EVO}^{+}	CO_{AUX}^{+}	0.0146★	61★	16★
4 d	Tetracycline	CO_{EVO}^{-}	CO_{AUX}^{-}	< 0.001	- 4.31	8
4 e	Tetracycline	CO_{EVO}^{+}	CO_{AUX}^{+}	< 0.001	- 7.66	8
4 d,e	Tetracycline	CO_{EVO}^{-}	CO_{EVO}^{+}	0.7452	- 1.01	8
4 d,e	Tetracycline	CO_{AUX}^{-}	CO_{AUX}^{+}	< 0.001	- 11.31	8
4 f	Tetracycline	CO_{EVO}^{-}	CO_{AUX}^{-}	0.2542★	159★	16★
4 f	Tetracycline	CO_{EVO}^{-}	CO_{EVO}^{+}	< 0.001★	6★	16★
4 f	Tetracycline	CO_{EVO}^{-}	CO_{AUX}^{+}	< 0.001★	0★	16★
4 f	Tetracycline	CO_{AUX}^{-}	CO_{EVO}^{+}	< 0.001★	1★	16★
4 f	Tetracycline	CO_{AUX}^{-}	CO_{AUX}^{+}	< 0.001★	0★	16★
4 f	Tetracycline	CO_{EVO}^{+}	CO_{AUX}^{+}	< 0.001★	31★	16★

Supplementary Note

Supplementary Note 1 | Statistical model used to analyse growth patterns during the evolution experiment.

Linear mixed models (LMM) were used with *bacterial growth* as the response variable. As the antibiotics chloramphenicol and tetracycline were applied in different concentrations (depending on their respective MIC, Fig. 4), two separate analyses were conducted: one for chloramphenicol and one for tetracycline (for each case: n = 320). As fixed effects, the *antibiotic concentration* and the *strain type in the presence or absence of amino acids* were included. We controlled for multiple measurements by including replicates as a random effect in the model.

Before running the models, the two numerical covariates were z-transformed to a mean of zero and a standard deviation of one, and the response variable were square root-transformed to achieve a more symmetrical distribution. Models were fitted in R[1] by using the function *lmer* from the R package lme4[2]. The LMMs were verified to test whether the assumptions of normal distribution and homogeneous residuals were fulfilled by visually inspecting a qqplot and plotting the residuals against the fitted values. In both models, no obvious deviation from the abovementioned assumptions were detected. Additionally, model stability was scrutinized by excluding each level of the random effect at a time from the data. A comparison of the model estimates derived for the reduced data set with those derived by the full data set ruled out the existence of overly influential cases. Variance Inflation Factors were calculated (VIF, [3]) using the function *vif* of the R-package car[4]. The results did not indicate collinearity to be an issue (largest VIF = 2.00). For LMMs, statistical significance of the full model was determined by comparing its fit with that of the null model comprising only the random effect, using a likelihood ratio test (LRT)[5]. For this, the R function *anova package stats* was used with argument test set to *Chisq*. The P-values for fixed effects were based on a likelihood ratio test, comparing the full model with a reduced model excluding the fixed effects[6, 7] using the R function *drop1* with argument test set to *Chisq*. To allow for an LRT, the models were fitted using Maximum Likelihood (rather than Restricted Maximum Likelihood[8]).

The results of the tests evaluating the models proposed revealed a clear influence of the fixed effects on the response variable (i.e. bacterial growth). LRT comparing full and null model for chloramphenicol: $\chi^2 = 93.999$, df = 7, P < 0.001 and tetracycline: $\chi^2 = 88.155$, df = 7, P < 0.001, Each fixed factor: a) antibiotic concentration and b) strain type in the presence or absence of amino acids, has a statistically significant effect on bacterial growth. For chloramphenicol, a: LRT = 24.185, P < 0.001, b: LRT = 77.625, P < 0.001. For tetracycline, a: LRT = 24.838, P < 0.001, b: LRT = 48.882, P < 0.001.

To compare between strain types in the presence or absence of amino acids, contrasts between growth levels were calculated using *emmeans* from the emmeans package function in R[9].

Supplementary References

1. RCoreTeam. R: A language and environment for statistical computing. *R Foundation for Statistical Computing, Vienna, Austria* https://www.R-project.org/ (2017).
2. Bates, D., Mächler, M., Bolker, B. & Walker, S. Fitting linear mixed-effects models using lme4. *J. Stat. Softw.* **67**, 1-48 (2013).
3. Field, A. *Discovering statistics using IBM SPSS statistics*, 4th edn. (SAGE Publications, London, 2013).
4. Fox, J. & Weisberg, S. Car: Companion to applied regression. *SAGE Publications* https://cran.r-project.org/web/packages/car/index.html (2011).
5. Dobson, A. & Barnett, A. Poisson regression and log-linear models. *An introduction to GLMs*, 165-171 (2008).
6. Barr, D. J., Levy, R., Scheepers, C. & Tily, H. J. Random effects structure for confirmatory hypothesis testing: Keep it maximal. *J. Mem. Lang.* **68**, 255-278 (2013).
7. Dobson, A. J. & Barnett, A. G. *An introduction to generalized linear models*, 4th edn. (CRC press, Boca Raton, 2018).
8. Bolker, B. M.*, et al.* Generalized linear mixed models: a practical guide for ecology and evolution. *Trends Ecol. Evol.* **24**, 127-135 (2009).
9. Lenth, R., Singmann, H., Love, J., Buerkner, P. & Herve, M. Emmeans: Estimated marginal means, aka least-squares means. *R package version* **1**, 3 (2018).
