# Supplementary material for: Obligate mutualistic cooperation limits evolvability
Source: Nat Commun. 2022 Jan 17;13:337. doi: 10.1038/s41467-021-27630-9 (PMC8764027; doi:10.1038/s41467-021-27630-9)
Supplement: Supplementary file 3 — Reporting Summary [file 41467_2021_27630_MOESM3_ESM.pdf]

nature research

Corresponding author(s): Christian Kost

Last updated by author(s): Nov 19, 2021

Reporting Summary

Nature Research wishes to improve the reproducibility of the work that we publish. This form provides structure for consistency and transparency in reporting. For further information on Nature Research policies, see our Editorial Policies and the Editorial Policy Checklist.

Statistics

For all statistical analyses, confirm that the following items are present in the figure legend, table legend, main text, or Methods section.

n/a	Confirmed	
☐	☒	The exact sample size (*n*) for each experimental group/condition, given as a discrete number and unit of measurement
☐	☒	A statement on whether measurements were taken from distinct samples or whether the same sample was measured repeatedly
☐	☒	The statistical test(s) used AND whether they are one- or two-sided<br>*Only common tests should be described solely by name; describe more complex techniques in the Methods section.*
☐	☒	A description of all covariates tested
☐	☒	A description of any assumptions or corrections, such as tests of normality and adjustment for multiple comparisons
☐	☒	A full description of the statistical parameters including central tendency (e.g. means) or other basic estimates (e.g. regression coefficient) AND variation (e.g. standard deviation) or associated estimates of uncertainty (e.g. confidence intervals)
☐	☒	For null hypothesis testing, the test statistic (e.g. *F*, *t*, *r*) with confidence intervals, effect sizes, degrees of freedom and *P* value noted<br>*Give P values as exact values whenever suitable.*
☒	☐	For Bayesian analysis, information on the choice of priors and Markov chain Monte Carlo settings
☒	☐	For hierarchical and complex designs, identification of the appropriate level for tests and full reporting of outcomes
☒	☐	Estimates of effect sizes (e.g. Cohen's *d*, Pearson's *r*), indicating how they were calculated

Our web collection on statistics for biologists contains articles on many of the points above.

Software and code

Policy information about availability of computer code

Data collection	No commercial, open source or custom code was used to collect the data in this study.
Data analysis	SPSS (Version 25, IBM®), R (Version 4.03), and Mathematica 12.0.0.0 were used for data analysis.

For manuscripts utilizing custom algorithms or software that are central to the research but not yet described in published literature, software must be made available to editors and reviewers. We strongly encourage code deposition in a community repository (e.g. GitHub). See the Nature Research guidelines for submitting code & software for further information.

Data

Policy information about availability of data

All manuscripts must include a data availability statement. This statement should provide the following information, where applicable:

- Accession codes, unique identifiers, or web links for publicly available datasets
- A list of figures that have associated raw data
- A description of any restrictions on data availability

The source data and code generated in this study are provided in the Zenodo database (accession number: https://zenodo.org/record/4895589#.YSjdIN9CRaR) and the generated strains are available from the corresponding author upon request.

Field-specific reporting

Ecological, evolutionary & environmental sciences study design

All studies must disclose on these points even when the disclosure is negative.

Study description	This study included two bacterial genotypes that derived from the bacterial progenitor strain Escherichia coli BW25113. Each strain was auxotrophic for one of two different amino acids (i.e. tryptophan (ΔtrpB) and tyrosine (ΔtyrA)) and originated from a previous study, where they have been experimentally coevolved. This selection regime led to an enhanced production of the reciprocally exchanged amino acids at a cost to the producing cells.<br>In the current study, an evolution experiment was performed in which the stains were cultivated in either monoculture or under coculture conditions. Monocultures of the tryptophan- and tyrosine-auxotrophic strains were supplemented with both tryptophan and tyrosine simultaneously (100 mM each). Over the course of the evolution experiment, concentrations of the four antibiotics used (i.e. ampicillin, chloramphenicol, kanamycin, and tetracycline) were gradually increased to exert an environmental selection pressure. The growth of all cultures was tracked by quantifying their population density (OD600nm) while propagating them to fresh medium every 72 h. Analyzing and comparing the resulting growth patterns of mono- and cocultures enabled us to draw inferences on the evolutionary trajectories of bacteria growing in obligate mutualistic cooperation compared to independent monocultures.
Research sample	The study included two auxotrophic bacterial genotypes Escherichia coli K12 BW25113 ΔtrpB ara- ΔLacZ and Escherichia coli K12 BW25113 ΔtyrA ara+ LacZ+ that were derived from a previous study, where they have evolved a reciprocal exchange of the focal amino acids at a cost to the producing cells. Each of the three tested consortia (i.e. tryptophan auxotroph monoculture, tyrosine auxotroph monoculture, and coculture of both auxotrophs) was subjected to the four different antibiotic treatments. Each consortium-treatment combination started with 16 replicates for the first two transfers (i.e. equilibration period) to reduce possible noise intoduced before starting the antibiotic treatment. Then we split those equilibrated cultures into 5 independent replicates, respectively, resulting in 80 replicates that were observed for the remaining 39 days (i.e. 13 time points) of the evolution experiment with a quantification of the cultures' absorbance at 600 nm every 72 h, while propagating them to fresh medium. In total, 1,088 data points were analyzed per consortium-treatment combination.
Sampling strategy	An experiment was performed with 80 replicates for each consortium-treatment combination. In our experiment, growth was consistently quantified as a continuous variable at a predefined time point (i.e. 72 h) after inoculation. With this approach, we were able to quantitatively compare the evolutionary trajectories of different samples.
Data collection	Growth data was recorded using a microplate reader (FilterMax F5, Molecular Devices) to measure the optical density of test cultures at a wavelength of 600 nm. The resulting data was copied into Microsoft Excel spreadsheets for subsequent analysis and further processed with R (Version 4.03) and Mathematica 12.0.0.0.
Timing and spatial scale	The evolution experiment lasted for 45 days (i.e. 25.09.2018 - 09.11.2018), and growth was quantified every 72 h, starting with the first inoculation t = 0 h (i.e. 16 individual time points). Previous experiments have shown that all tested cultures have entered stationary growth phase at this time point, corresponding to the maximum optical density (OD600nm) that the bacterial consortia will be physiologically able to achieve. The experiment was performed using commercially available 96 deep-well plates (Thermo Scientific Nunc).
Data exclusions	No data was excluded from any of the analyses shown in our manuscript.
Reproducibility	Each consortium-treatment combination was independently replicated 80-times.
Randomization	It was not possible to randomize the inoculation of strains into culture wells, because a random distribution of the samples would have impeded the realization of the evolution experiment. Because growth conditions within a single plate are nearly identical for each well, we instead treated each replicate plate (i.e. 5 per treatment) as one experimental block to account for variation that may have existed between plates.
Blinding	During the acquisition of the experimental data, the performing scientist did not know which exact sample he analyzed at that point in time. Plates have been numbered to prevent any unconscious bias. Only when the resulting data was further analyzed, the differentially numbered plates were translated back to assign them to their exact identity of samples.

Did the study involve field work? ☐ Yes ☒ No

Reporting for specific materials, systems and methods

We require information from authors about some types of materials, experimental systems and methods used in many studies. Here, indicate whether each material, system or method listed is relevant to your study. If you are not sure if a list item applies to your research, read the appropriate section before selecting a response.

Materials & experimental systems

n/a	Involved in the study
☒	☐ Antibodies
☒	☐ Eukaryotic cell lines
☒	☐ Palaeontology and archaeology
☒	☐ Animals and other organisms
☒	☐ Human research participants
☒	☐ Clinical data
☒	☐ Dual use research of concern

Methods

n/a	Involved in the study
☒	☐ ChIP-seq
☒	☐ Flow cytometry
☒	☐ MRI-based neuroimaging
